# Concurrent Malignant Solitary Fibrous Tumor Arising from the Omentum and Grade 3 Endometrial Endometrioid Adenocarcinoma of the Uterus with *p53* Immunoreactivity

**DOI:** 10.1155/2014/216340

**Published:** 2014-07-10

**Authors:** Naoya Harada, Ichiro Nobuhara, Noriko Haruta, Yumi Higashiura, Hideki Watanabe, Sumire Ohno

**Affiliations:** Department of Obstetrics and Gynecology, Nara City Hospital, 1-50-1 Higashikidera-cho, Nara, Nara Prefecture 630-8305, Japan

## Abstract

A malignant solitary fibrous tumor arising from the omentum is extremely rare. To our knowledge, this is the first case of a malignant solitary fibrous omentum tumor coexisting with uterine corpus cancer. A 62-year-old woman presented to our hospital with vaginal discharge. Endometrioid adenocarcinoma was diagnosed by endometrial curettage. In addition, a solid tumor in front of the uterus was detected following computed tomography and/or magnetic resonance imaging, which was suspected to be a primary (or secondary) malignant tumor arising from the omentum. Hysterectomy, bilateral salpingo-oophorectomy, omentectomy, and lymphadenectomy were performed. A malignant solitary fibrous tumor of the omentum and grade 3 endometrioid adenocarcinoma of the uterus were diagnosed by pathohistological analysis. Interestingly, the tumor cells were immunoreactive for *p53*. Adjuvant chemotherapy was administered for the uterine corpus cancer and the patient remains healthy 48 months after the surgery. These tumors may have become malignant due to the presence of *p53* mutations.

## 1. Introduction 

A solitary fibrous tumor (SFT) is a rare neoplasm mainly originating in the pleura; however, extrathoracic SFTs have been increasingly described, such as those in the meninges, liver, upper respiratory tract, orbit, thyroid, salivary gland, and female genital tract [1]. SFT of the peritoneum, particularly one arising in the lesser omentum, is extremely rare [2]. Moreover, although SFTs are usually benign, malignant SFTs have been described in a limited number of reports [3–8]. To our knowledge, a case of concurrent malignant SFT arising from the omentum and grade 3 endometrial endometrioid adenocarcinoma of the uterus has not been reported previously. Interestingly, the tumor cells were immunoreactive for* p53*. We present here the clinical course and immunohistological findings.

## 2. Case Presentation

**Figure 1:**
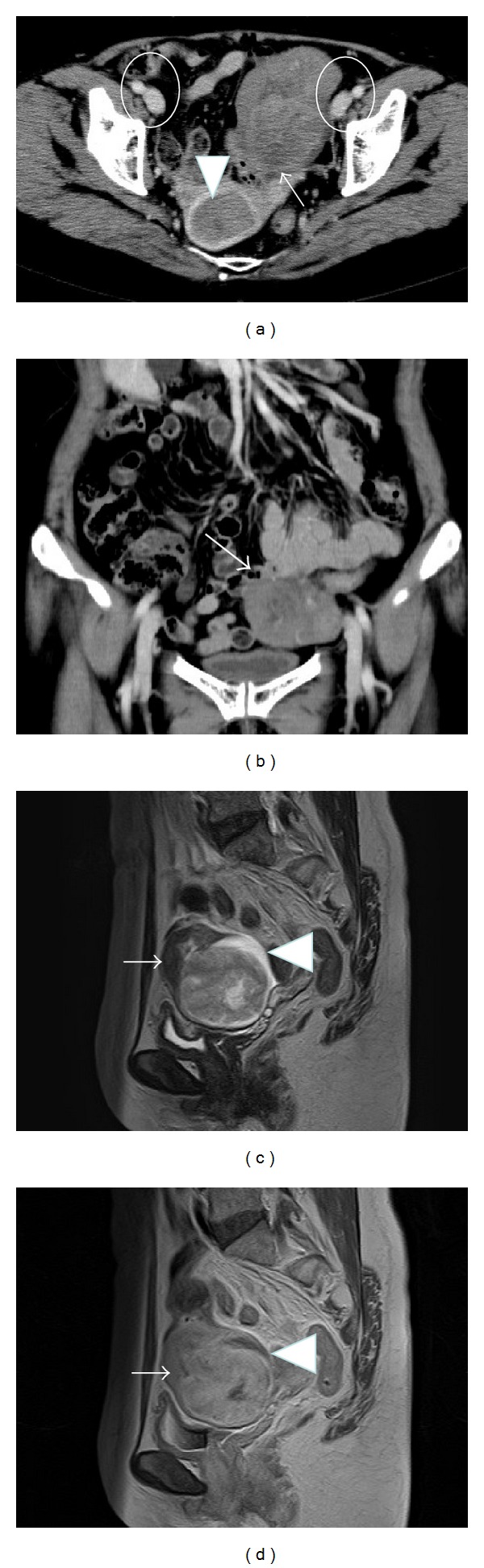
Axial (a) and coronal (b) computed tomography and sagittal magnetic resonance imaging (c, d). (a) Two tumors were detected in both the uterus (triangle) and the peritoneal cavity (arrow). The bilateral ovaries (circle) could be observed. (b) The tumor in the peritoneal cavity originated from the omentum (arrow). (c) The tumor in the peritoneal cavity heterogeneously exhibited an isointense-to-hyperintense signal on T2-weighed images. (d) The tumor was prominently enhanced on contrast-enhanced T1-weighted images. The flow void from the omentum to the tumor (arrow) and ascites (triangle) could also be detected.

**Figure 2:**
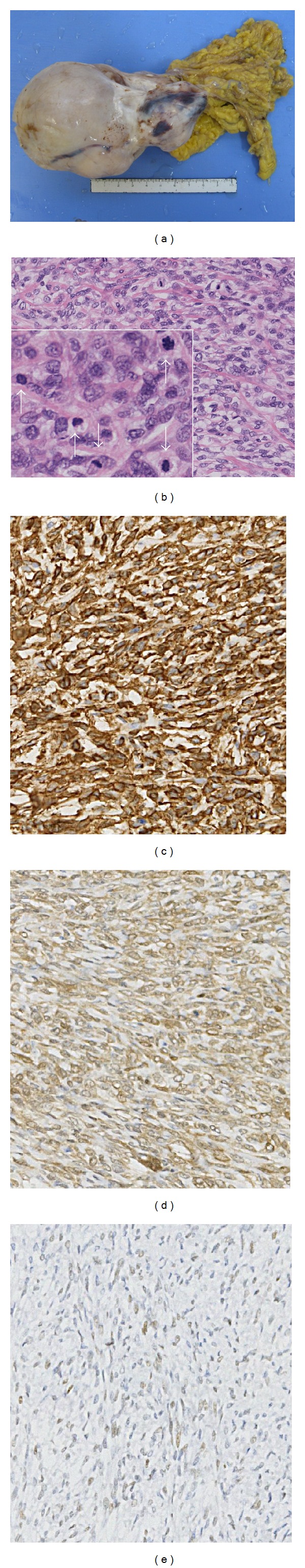
Macroscopic and microscopic findings of the tumor in the peritoneal cavity. (a) The tumor measured approximately 10 cm in the peritoneal cavity and originated from the omentum. The cut surface was milky white and smooth. (b) The tumor was hypercellular and composed of atypical tumor cells with a high mitotic activity (hematoxylin and eosin (HE) stain, objective magnification ×20). Inset: large magnification view of the tumor cells with hyperchromatic nuclei and increased mitotic activity (arrow; HE stain, objective magnification ×40). (c) The tumor cells were strongly positive for CD34 (objective magnification ×20). (d) The tumor cells were moderately positive for* p16* (objective magnification ×20). (e) The tumor cells were weakly positive for* p53* (objective magnification ×20).

A 62-year-old menopausal woman presented to our hospital with vaginal discharge. The uterus, which was slightly larger than a hen's egg, along with a movable goose-egg-sized hard tumor in front of the uterus, was palpable during the internal examination. Grade 3 endometrioid adenocarcinoma of the uterus was detected by endometrial curettage. Computed tomography (CT) and/or magnetic resonance imaging (MRI) detected 2 solid tumors in both the uterus and the peritoneal cavity, measuring approximately 4 cm and 10 cm, respectively. Bilateral ovaries could also be detected (Figure 1(a)). The tumor in front of the uterus originated from the omentum on coronal CT (Figure 1(b)). On MRI, the tumors exhibited a hypointense signal on T1-weighted images (T1WI) and a heterogeneous isointense-to-hyperintense signal on T2-weighted images (Figure 1(c)). They were prominently enhanced on contrast-enhanced T1WI (Figure 1(d)), and high-intensity areas were visible in the diffusion-weighted images. The flow void from the omentum to the tumor could be also detected. Preoperatively, these results were highly suggestive of uterine corpus cancer and a primary (or secondary) malignant tumor of the omentum. The patient's serum CA125 and serum CA19-9 levels were 38.7 U/mL and 35.1 U/mL, respectively. As expected, a laparotomy revealed that the tumor in the peritoneal cavity originated from the omentum, and it was well circumscribed with a gross maximum dimension of 10 cm (Figure 2(a)). It was diagnosed as a sarcoma by pathohistological analysis during surgery, and then a hysterectomy, bilateral salpingo-oophorectomy, omentectomy, and lymphadenectomy were performed. Histological examination revealed that the tumor in the uterus was a grade 3 endometrial endometrioid adenocarcinoma (pT1bN0M0). The tumor cells were strongly immunoreactive for* p53*. Tumor in the peritoneal cavity was hypercellular and exhibited a patternless arrangement of fibroblastic spindle cells. It was composed of markedly atypical cells with a high mitotic activity (>10 mitotic figures/10 high power fields (HPF); Figure 2(b)) and the proliferative index as assessed by Ki-67 labeling index positivity was >30%. Immunohistochemical staining demonstrated that the tumor cells were positive for vimentin, bcl-2, CD34 (Figure 2(c)), CD99, CD10, S-100, c-kit, epithelial membrane antigen, cytokeratin AE1/AE3, progesterone receptor,* p16* (Figure 2(d)), and* p53* (Figure 2(e)), according to various grades of intensity (Table 1). They were negative for smooth muscle actin, desmin, D2-40, calretinin, estrogen receptor, and CD31. Therefore, we determined that the omentum tumor was a malignant SFT with sarcomatous growth. Six cycles of adjuvant chemotherapy (paclitaxel, 175 mg/m^2^ and carboplatin, AUC 5) were administered for the uterine corpus cancer. The patient remains healthy 48 months after the surgery.

## 3. Discussion

SFT is a rare neoplasm that is thought to originate from submesothelial mesenchymal cells [3]. SFT often arises in the pleura, but may also arise in different extrapleural sites. Generally, tumors of the lesser omentum are rare, and few reports have described SFT arising from the omentum [2, 4]. To our knowledge, this is the first case of a malignant SFT originating from the omentum complicated with uterine corpus cancer. Accurate diagnosis of this type of tumor is difficult and, in our case, although our findings were highly suggestive of a primary (or secondary) malignant tumor of the omentum, malignant SFT could not be diagnosed preoperatively.

England et al. [5] considered SFT to be malignant if the following histologic features were present: high cellularity, >4 mitotic figures/10 HPF, pleomorphism, hemorrhage, and necrosis. These criteria are used worldwide to distinguish between benign SFT and malignant SFT [3]. The Ki-67 labeling index is also diagnostically relevant in the evaluation of malignant SFT and Sun et al. [6] thought that it was also useful as a prognostic indicator. In our case, as England's criteria were completely fulfilled and the Ki-67 labeling index positivity was extremely high, malignant SFT was diagnosed.

At present, based upon the poor existing data, the optimal therapy for malignant SFT is uncertain and the assessment of the most effective means of management is difficult [4, 8]. While awaiting to acquire a wider clinical experience on this rare form of omental tumor, Patrelli et al. [9] recommend a “customized” treatment that uses surgery and either neoadjuvant or adjuvant chemo/radiation therapy, which, in the event of early diagnosis, can be used to achieve a disease-free result. Poor prognostic markers of SFT include positive surgical margins, tumor size measuring >10 cm, and mitotic activity of 10 mitotic figures/10 HPF [2]. In our case, complete excisional surgery for malignant SFT was carried out, so adjuvant chemotherapy only for the uterine corpus cancer was administered, but long-term careful follow-up is necessary.

Other soft tissue neoplasia, such as synovial sarcoma, fibrosarcoma, malignant peripheral nerve sheath tumor, hemangiopericytoma, hemangioendothelioma, angiosarcoma, leiomyosarcoma, endometrial stromal sarcoma, and a gastrointestinal stromal tumor should be considered in the histopathological differential diagnosis of STF. Immunohistochemical staining features are very useful for SFT diagnosis. In our case, these features were evaluated by intensity (strongly positive, moderately positive, weakly positive, and negative) and are summarized in Table 1. The diagnosis of SFT has been refined by the availability of immunohistochemical markers such as CD34, vimentin, bcl-2, and CD99 [4]. Moreover, as CD10, S-100, and c-kit were weakly immunoreactive in this patient, malignant SFT with sarcomatous growth was diagnosed. Synovial sarcoma, fibrosarcoma, and malignant peripheral nerve sheath tumors are usually CD34 negative. Hemangiopericytoma, hemangioendothelioma, and angiosarcoma are usually CD31 positive. Leiomyosarcoma and gastrointestinal stromal tumors will be positive for at least one of the myogenic immunohistochemical stains (usually smooth muscle actin or desmin), while endometrial stromal sarcoma will be immunoreactive for the estrogen receptor.

The* p53* tumor suppressor gene plays an important role in the regulation of cell growth. Mutations in this gene have been reported in various malignant tumors and are thought to be involved in pathogenesis and progression [7]. In relation to the gynecological organs,* p53* mutations are known to be associated with uterine corpus cancer development (grade 3 endometrial endometrioid adenocarcinoma) [10]. However, the mechanism of malignant SFT development has not yet been fully elucidated [7]. There have been few reports examining* p53* expression in SFT [4, 7]. In our case, the malignant SFT cells were moderately positive for* p16* and weakly positive for* p53*. Mosquera and Fletcher suggested that* p53* and* p16* overexpression in the anaplastic component of SFT are in keeping with their potential role in the dedifferentiation process previously identified in some sarcomas and carcinomas [4]. Yokoi et al. suggested that malignant SFTs develop mainly in 2 ways: malignant transformation within benign SFT and the* de novo* occurrence of malignant SFT [7]. Our case of a concurrent malignant SFT arising in the omentum and grade 3 endometrial endometrioid adenocarcinoma of the uterus may have occurred* de novo* due to* p53* mutations.

## Figures and Tables

**Table 1:** Immunohistochemical staining features.

Strongly positive	vimentin, bcl-2, and CD34
Moderately positive	*p16* and CD99
Weakly positive	*p53*, CD10, progesterone receptor, S-100, c-kit, EMA, and cytokeratin AE1/AE3
Negative	smooth muscle actin, desmin, D2-40, calretinin, estrogen receptor, and CD31

EMA: epithelial membrane antigen.
